# Efficacy of limb salvage with primary tumor resection simultaneously for solitary bone metastasis in limbs

**DOI:** 10.1186/s12957-016-0786-8

**Published:** 2016-02-04

**Authors:** Dong-dong Cheng, Jie-lai Yang, Tu Hu, Qing-cheng Yang

**Affiliations:** Department of Orthopeadics, Shanghai Jiao Tong University Affiliated Sixth People’s Hospital, No.600, Yishan Road, Shanghai, 200233 China

**Keywords:** Solitary bone metastasis, Limb salvage, Limb function, Life quality, Regional recurrence

## Abstract

**Background:**

This study aims to evaluate the efficacy of limb salvage with primary tumor resection on patients with solitary bone metastasis.

**Methods:**

A retrospective treatment outcome review was performed on 20 patients with solitary bone metastasis as the primary clinical symptom who were admitted to the hospital between 2006 and 2010. With primary tumor resection, 18/20 patients received limb salvage surgery simultaneously. Pain scoring was assessed using the 0 to 10 numerical rating scale. The quality of life scoring was performed before and 3 months after surgery using the SF-30 scoring system. In addition, limb function was assessed 3 months after the operation using the Scoring System of American Musculoskeletal Tumor Society system (MSTS).

**Results:**

The pain symptom was significantly ameliorated after the operation (*t* = 26.653, *P* < 0.001), and the quality of life dramatically improved (*t* = −20.581, *P* < 0.001). The postoperative MSTS scores ranged from 18 to 27. The average score was 23.10 ± 2.36. The Kaplan-Meier analysis showed that no significant differences (*χ*2 = 1.589, *P* = 0.207) were observed in the tumor-free survival time between the wide and marginal resections.

**Conclusions:**

The application of the wide or marginal excision for the primary lesion and bony metastasis focus, based on the principles of primary bone tumors, can significantly relieve the pain and improve the quality of life and limb function of patients whose solitary bone metastasis was manifested as the first sign.

**Electronic supplementary material:**

The online version of this article (doi:10.1186/s12957-016-0786-8) contains supplementary material, which is available to authorized users.

## Background

In metastatic cancer, the bone is one of the most likely metastatic sites just following the lung and the liver. Bone metastases often occur in some cancers, including breast cancer, prostate cancer, kidney cancer, thyroid cancer, and lung cancer, of which breast cancer and prostate cancer take the lead [1]. Seventy percent of bone metastases occur in the axial skeleton, while 10 % in the limbs. Limb bone metastases occur in the proximal long bones, such as the proximal humerus and the proximal femur [2]. Patients with bone metastases often experience severe complications, including pain, limited mobility, hypercalcemia, pathologic fracture, spinal cord compression, or nerve root compression, which will seriously affect the patient’s quality of life, thereby affecting the treatment of the primary tumor [3]. In cancer patients, emergence of bone metastases usually implies that the diseases are in the middle or final term. Therefore, improving the quality of life, alleviating local symptoms, and curing relative complications are the primary purposes for bone metastases.

As reported in the previous literature, the general surgical treatment of limb bone metastases was amputation [4]. But now, the most well accepted therapy is the palliative surgical treatment or the limb salvage surgery including chemotherapy, radiotherapy, bisphosphonate, and analgesic drug therapy [5]. For bone metastases in limbs, palliative surgery mainly aims to treat or lower the risk of the pathologic fracture of the long bone and the hip. The primary surgical approaches include plate fixation, large segment resection and reconstruction, intramedullary nails, and external fixation. Hardman et al. reported that taking the preventive internal fixation treatment for limb bone metastases has more favorable effort in prolonging survival and improving the quality of life than that after pathological fracture [6]. Studies have shown that patients with solitary bone metastasis have a better prognosis than those with multiple ones [7]. Therefore, for patients whose solitary bone metastasis was manifested as the first sign, wide or marginal excision for the primary lesion and bony metastasis focus according to treatment principles of the primary bone tumor can improve the patients’ quality of life. This group of patients received limb salvage surgery with primary tumor resection simultaneously. To analyze the efficacy of this strategy, the pain, quality of life, limb function, tumor-free survival time, and postoperative complications were evaluated.

## Methods

### Patients

From October 2006 to August 2010, 20 patients whose solitary bone metastasis was manifested as the first sign were treated in our hospital. The basic information of the 20 patients was shown in Table 1. The more detailed information of the patients was shown in Additional file 1: Table S1. There were 9 men and 11 women. The average age was 58.7 years (ranged 43–70 years). The follow-up time ranged from 6 to 30 months; the mean time is 14.5 months. The metastatic sites are located in the femur (*n* = 11), humerus (*n* = 7), and tibia (*n* = 2). The primary cancers involved lung cancer (*n* = 8), breast cancer (*n* = 3), kidney cancer (*n* = 4), prostate cancer (*n* = 2), thyroid cancer (*n* = 1), cervical cancer (*n* = 1), and gastric cancer (*n* = 1). The complaints included simple pain in 11 patients, pain and bump in 2, and pathological fracture in 6 patients. All the patients were admitted to hospital for pain at the site of bone metastases, local mass, or pathologic fractures as the first clinical manifestation, and further examination revealed the primary tumor. Primary tumor and bone metastases were confirmed by pathological examination. Written informed consent was obtained from the patients, and the study was performed in accordance with the Declaration of Helsinki and approved by the ethics committee of the Sixth People’ hospital of Shanghai Jiao Tong University.

**Table 1:** Basic information of the 20 patients

Case	Gender	Age	Metastatic position	Primary tumor	Surgical boundary	Preoperative pain score	Postoperative pain score	MSTS score	Preoperative QOL score	Postoperative QOL score	Follow-up (months)	Tumor-free survival time (months)
1	Male	43	Proximal femur	Lung cancer	Marginal	8	1	25	34	72	13	9
2	Male	56	Proximal femur	Prostate cancer	Wide	10	2	24	28	61	18	18
3	Female	46	Proximal humerus	Lung cancer	Wide	7	1	23	45	82	16	13
4	Female	45	Proximal humerus	Kidney cancer	Wide	9	2	22	32	65	30	30
5	Female	55	Middle humerus	Lung cancer	Wide	7	1	24	34	69	12	12
6	Male	68	Proximal femur	Lung cancer	Marginal	8	1	19	23	51	9	7
7	Female	67	Proximal femur	Cervical cancer	Wide	10	1	23	28	63	11	9
8	Female	55	Middle humerus	Lung cancer	Wide	6	1	27	37	72	6	5
9	Female	65	Proximal humerus	Breast cancer	Marginal	8	2	26	38	65	10	10
10	Female	55	Proximal humerus	Thyroid cancer	Wide	7	1	22	68	82	19	19
11	Female	58	Proximal tibia	Breast cancer	Marginal	9	1	25	30	63	14	14
12	Male	64	Distal femur	Kidney cancer	Marginal	8	3	24	33	60	16	12
13	Male	69	Proximal femur	Prostate cancer	Wide	8	1	23	41	67	14	11
14	Male	67	Proximal femur	Lung cancer	Wide	9	3	20	21	43	7	4
15	Male	58	Proximal tibia	Kidney cancer	Marginal	7	2	18	25	57	17	12
16	Female	45	Proximal humerus	Lung cancer	Marginal	10	2	23	34	68	14	9
17	Female	70	Proximal femur	Breast cancer	Wide	8	2	21	39	58	25	21
18	Male	66	Proximal femur	Gastric cancer	Marginal	7	1	25	45	68	17	17
19	Male	63	Proximal femur	Kidney cancer	Marginal	7	1	26	42	68	13	11
20	Female	59	Distal femur	Lung cancer	Wide	9	2	22	41	70	9	9

*QOL* quality of life

### Surgical procedures

Solitary bone metastasis was manifested as the first sign in all the patients in our group. Preoperative closed biopsy of bone lesion confirmed it was metastasis. In combination with other organ lesions, the patients were diagnosed as primary tumor associated with solitary bone metastases in the limbs. Eighteen patients underwent limb salvage surgery together with the primary tumor resection simultaneously according to the treatment principle of primary bone tumor under same anesthesia. Of two cases of prostate cancer, the patients were admitted to hospital because of pathological fracture of the proximal femur. The patient received proximal femoral tumor resection and prosthetic replacement, and the endocrine therapy was carried out after surgery. The other patient received bone tumor resection and prosthetic replacement, accompanying with the castration therapy. According to the Enneking Staging System, wide resection was done in 11 patients and marginal resection in 9 patients. The diagnosis was confirmed by pathological examination postoperatively. Perioperative complications were treated. The patients underwent adjuvant systemic chemotherapy or (and) local radiotherapy.

### Follow-up

Pain scoring and quality of life scoring were carried out preoperatively and postoperatively. Pain assessment was conducted using the 0 to 10 numerical rating scale preoperatively and 1 month after surgery. The quality of life scoring was performed preoperatively and 3 months after surgery using the SF-36 scoring system [8]. All ratings were done by the same person. Limb function score was evaluated using Musculoskeletal Tumor Society scoring system (MSTS score) 3 months after surgery [9].

### Statistical evaluation

The data were compiled and analyzed using SPSS version 21.0. Continuous data were expressed as mean values and standard deviation. Comparisons between different time points were done using paired Student’s *t* test. The Kaplan-Meier analysis was employed for survival analysis between groups. Tumor-free survival time is the period from surgery to the presence of new lesions. A significant result was taken as *p* < 0.05.

## Results

### Surgical complications

The average follow-up period was 14.5 months (ranged from 6 to 30 months). No patient was default in follow-up. Superficial incision infection was found in one patient, who was cured after debridement. Deep vein thrombosis was found in one patient and resolved after systemic anticoagulation therapy for 1 week. Pulmonary infection was detected in one patient who was treated by antibiotic therapy.

### Result of evaluation

As shown in Table 2, the mean preoperative pain score was 8.10 ± 1.17, whereas the postoperative pain score was 1.55 ± 0.69. The symptom was significantly ameliorated after the operation (*t* = 26.653, *P* < 0.001). The mean preoperative quality of life score was 35.90 ± 10.26, whereas the mean postoperative score was 65.20 ± 9.14. Therefore, the quality of life dramatically improved (*t* = −20.581, *P* < 0.001). The postoperative MSTS scores ranged from 18 to 27. The average score was 23.10 ± 2.36. All patients recovered the ability of daily living and could within walk with or without the aid of walker 1 month after surgery.

**Table 2:** Pre- and postoperative pain and quality of life scores

	Preoperative	Postoperative	*t*	*p*
Pain score	8.10 ± 1.17	1.55 ± 0.69	26.653	<0.001
Quality of life score	35.90 ± 10.26	65.20 ± 9.14	−20.581	<0.001

### Tumor-free survival time

**Fig. 1:**
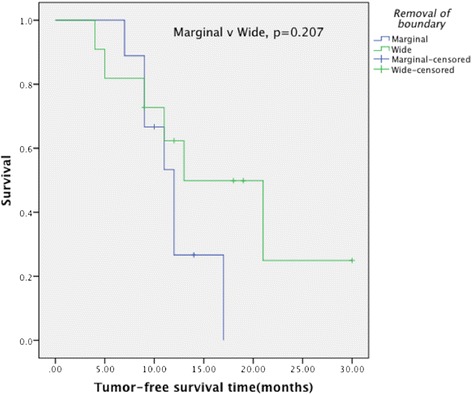
The comparison of tumor-free survival time between wide resection and marginal resection

In our group, 11 patients received wide resection and 9 patients received marginal resection. As shown in Fig. 1, the Kaplan-Meier analysis revealed that there was no significant difference between wide resection and marginal resection in tumor-free survival time (*χ*2 = 1.589, *P* = 0.207).

## Discussion

Metastatic bone cancer is a common malignancy. Most malignant tumors involve in bone metastases, 80 % of breast, prostate, and lung cancer are in the process [10]. In our study, the primary cancers involved lung cancer (*n* = 8), breast cancer (*n* = 3), kidney cancer (*n* = 4), prostate cancer (*n* = 2), thyroid cancer (*n* = 1), cervical cancer (*n* = 1), and gastric cancer (*n* = 1). Nowadays, the treatment of bone metastases is a comprehensive therapy. Although radiation therapy play a role in pain control and prevention of pathological fractures, surgery is more effective in functional recovery and prevention of the progression of local tumor [11]. Moreover, patients with bone metastases are usually older; they can hardly withstand the long-term and highly toxic chemotherapy. Thus, moderate surgery has a positive significance for patients with bone metastases. Surgical approaches vary in different patients. Factors affecting the surgical methods include age, systemic condition, and extent of bone metastases, the existence of metastases outside the bone, as well as the patient’s wishes [12].

**Fig. 2:**
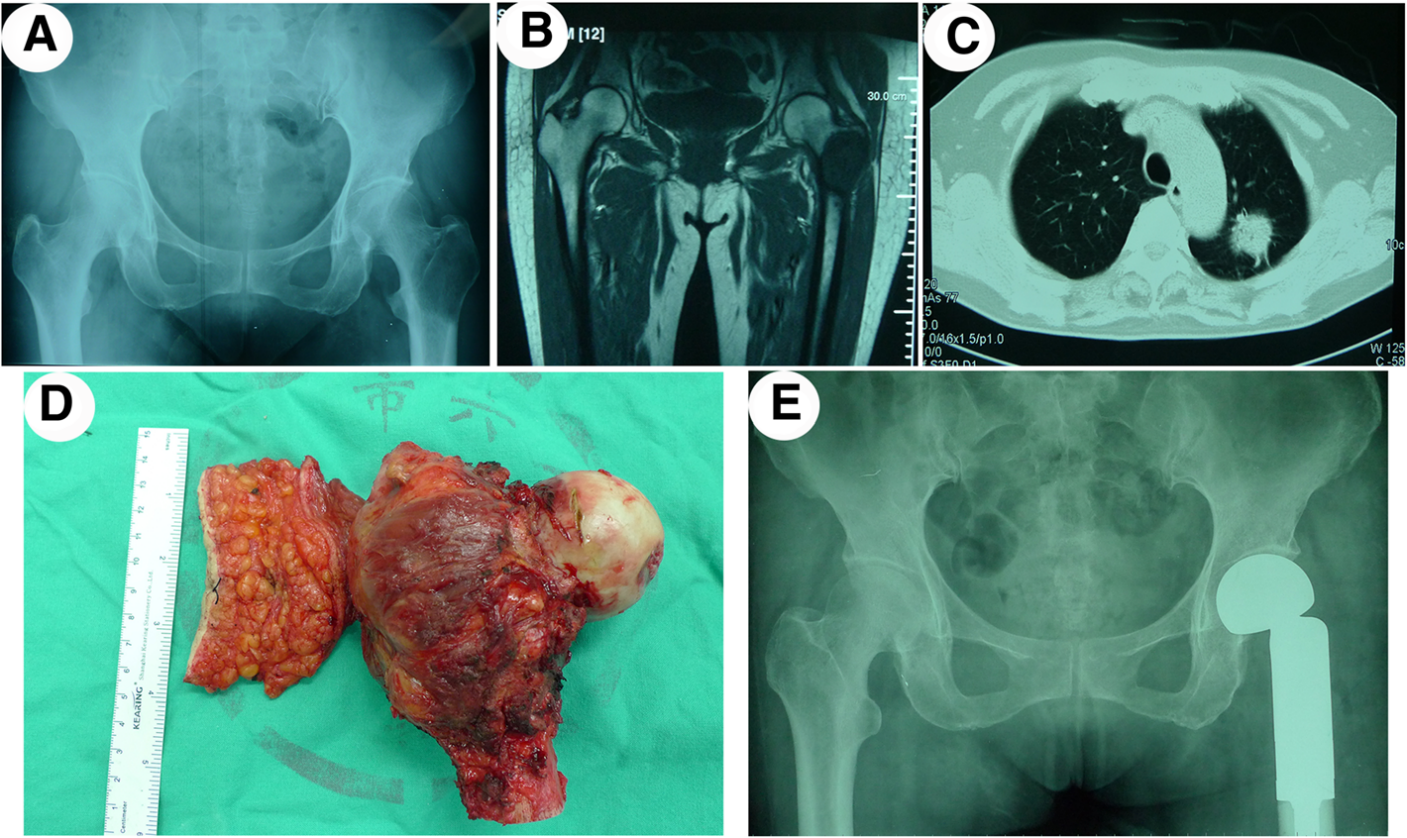
Typical case 1. **a** X-ray revealed bone destruction at the proximal end of the left femur; **b** MRI demonstrated hypointense signal in T1-weighted images; **c** CT revealed right lung cancer; **d** postoperative gross observation; **e** postoperative X-ray

**Fig. 3:**
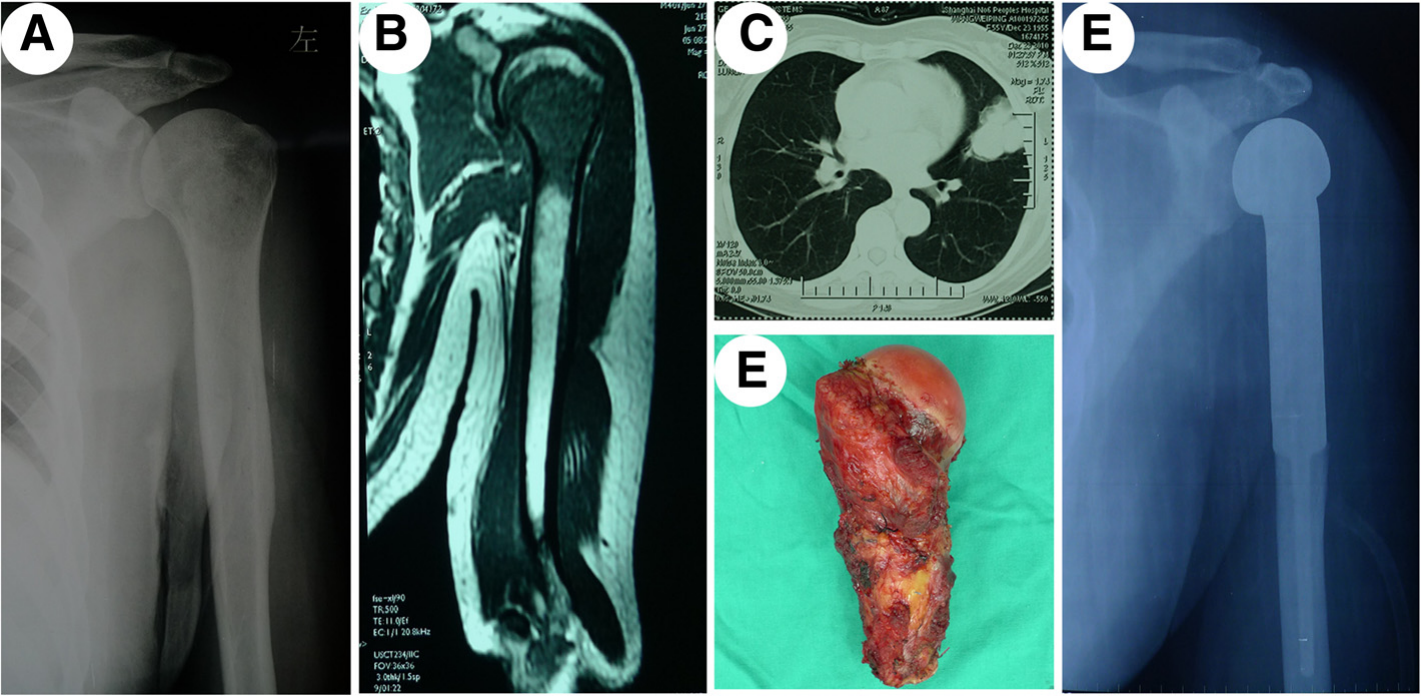
Typical case 2. **a** X-ray revealed bone destruction at the proximal end of the left humerus; **b** MRI demonstrated hypointense signal in T1-weighted images; **c** CT revealed right lung cancer; **d** postoperative gross observation; **e** postoperative X-ray

Bone metastases are the most common first manifestation of lung cancer, kidney cancer, myeloma, and lymphoma. Although breast and prostate cancer are the most common primary sites of bone metastases, they rarely take bone metastases as the first manifestation [13]. However, in our group, bone metastases were recognized as the first manifestation in all patients, and further examination revealed the primary tumor. For metastatic cancer patients without pathological fracture, it is advisable to receive non-surgical treatment. While for most patients with pathological fracture, limb salvage surgery is a preferable choice [14]. Pathological fracture occurs mainly in the proximal end of the bone and the backbone (Fig. 2). Therefore, the surgical fixation methods vary according to the metastatic tumor site. For fracture adjacent to the joint, tumor resection, and prosthetic replacement are mainly used. Six patients with pathological fracture adjacent to the joint underwent tumor resection and prosthetic replacement. When the fracture locates in the backbone, bone cement filling with intramedullary nailing is the better choice [15]. For bone metastases, patients without pathological fracture, Mirels et al. considered that if a total Mirels score of 8 or greater was identified, it was advisable to take measures to prevent the occurrence of pathologic fractures. If the Mirels score was greater than 9, the risk of pathological fractures rises to 33 %, it is necessary to take preventive internal fixation [16]. Studies have shown that, in the lung cancer, patients with bone metastases, limb salvage surgery with primary tumor resection simultaneously could significantly improve their survival time and prognosis (Fig. 3) [17, 18]. In the 20 patients of this group, bone metastases were recognized as the first performance of clinical manifestations, and further examination revealed the primary lesion. Among them, 18 received salvage treatment, consisting of tumor resection and prosthetic replacement, simultaneously with the resection of the primary tumor. For prostate cancer patients, one patient received endocrine therapy after limb salvage surgery; the other was given castration therapy. Pain was alleviated, and the quality of life was improved significantly after surgery.

In this group, 11 patients received wide resection of metastatic tumor, while 9 patients received marginal resection together with postoperative radiotherapy as an adjuvant therapy, which also received good control of the local tumor. No local recurrence was observed in this group. And there was no significant difference between the patients with wide resection and those with marginal resection in their tumor-free survival time. Bauer reported that in Karolinska hospital, nine kidney cancer patients with solitary bone metastases underwent wide or marginal resection with no local recurrence or implant failures during follow-up [13]. Similarly, as reported in literature, the presence of primary cancer and poor performance status were identified as independent prognostic factors using Cox regression multivariate analysis; however, surgical margin was not significantly related to patient survival [19]. Cho also showed that wide resection conveyed no survival advantage over curettage in patients with solitary bone metastasis from hepatocellular carcinoma [20]. Therefore, wide or marginal tumor resection is not the major factor in determining the tumor-free survival time.

## Conclusions

In short, for the bone metastases, patients whose solitary bone metastasis was manifested as the first sign, limb salvage surgery with primary tumor resection simultaneously according to the treatment principles of primary bone cancer can reduce the patients’ pain and improve their quality of life, without increasing the rate of local recurrence. However, as the patients were followed up in a relative short time, the impact of limb salvage therapy on patients’ overall survival needs further follow-up.

### Consent

Written informed consent was obtained from the patient’s guardian/parent/next of kin for the publication of this report and any accompanying images.

## Additional file

Additional file 1: Table S1. Additonal information of 20 patients. (DOCX 18 kb)

## References

[1] Riccio AI, Wodajo FM, Malawer M (2007). Metastatic carcinoma of the long bones. Am Fam Physician.

[2] Hage WD, Aboulafia AJ, Aboulafia DM (2000). Incidence, location, and diagnostic evaluation of metastatic bone disease. Orthop Clin North Am.

[3] Coleman RE (2001). Metastatic bone disease: clinical features, pathophysiology and treatment strategies. Cancer Treat Rev.

[4] Mundy GR (2002). Metastasis to bone: causes, consequences and therapeutic opportunities. Nat Rev Cancer.

[5] Harrington KD (1995). Orthopaedic management of extremity and pelvic lesions. Clin Orthop Relat Res.

[6] Hardman PD, Robb JE, Kerr GR, Rodger A, MacFarlane A (1992). The value of internal fixation and radiotherapy in the management of upper and lower limb bone metastases. Clin Oncol (R Coll Radiol).

[7] Koizumi M, Yoshimoto M, Kasumi F, Ogata E (2003). Comparison between solitary and multiple skeletal metastatic lesions of breast cancer patients. Ann Oncol.

[8] Reulen RC, Zeegers MP, Jenkinson C, Lancashire ER, Winter DL, Jenney ME (2006). The use of the SF-36 questionnaire in adult survivors of childhood cancer: evaluation of data quality, score reliability, and scaling assumptions. Health Qual Life Outcomes.

[9] Enneking WF, Dunham W, Gebhardt MC, Malawar M, Pritchard DJ (1993). A system for the functional evaluation of reconstructive procedures after surgical treatment of tumors of the musculoskeletal system. Clin Orthop Relat Res.

[10] Janjan N (2001). Bone metastases: approaches to management. Semin Oncol.

[11] Russo P (2000). Renal cell carcinoma: presentation, staging, and surgical treatment. Semin Oncol.

[12] Fottner A, Szalantzy M, Wirthmann L, Stähler M, Baur-Melnyk A, Jansson V (2010). Bone metastases from renal cell carcinoma: patient survival after surgical treatment. BMC Musculoskelet Disord.

[13] Bauer HC (2005). Controversies in the surgical management of skeletal metastases. J Bone Joint Surg (Br).

[14] Healey JH, Brown HK (2000). Complications of bone metastases: surgical management. Cancer.

[15] Coleman RE, Lipton A, Roodman GD, Guise TA, Boyce BF (2010). Metastasis and bone loss: advancing treatment and prevention. Cancer Treat Rev.

[16] Mirels H (2003). Metastatic disease in long bones: a proposed scoring system for diagnosing impending pathologic fractures. 1989. Clin Orthop Relat Res.

[17] Hirano Y, Oda M, Tsunezuka Y, Ishikawa N, Watanabe G (2005). Long-term survival cases of lung cancer presented as solitary bone metastasis. Ann Thorac Cardiovasc Surg.

[18] Agarwala AK, Hanna NH (2005). Long-term survival in a patient with stage IV non-small-cell lung carcinoma after bone metastasectomy. Clin Lung Cancer.

[19] Hoshi M, Takada J, Ieguchi M, Takahashi S, Nakamura H (2013). Prognostic factors for patients with solitary bone metastasis. Int J Clin Oncol.

[20] Cho HS, Oh JH, Han I, Kim HS (2009). Survival of patients with skeletal metastases from hepatocellular carcinoma after surgical management. J Bone Joint Surg (Br).

