# Supplementary material for: Efficacy of limb salvage with primary tumor resection simultaneously for solitary bone metastasis in limbs
Source: World J Surg Oncol. 2016 Feb 4;14:31. doi: 10.1186/s12957-016-0786-8 (PMC4743159; doi:10.1186/s12957-016-0786-8)
Supplement: Additional file 1: Table S1. — Additonal information of 20 patients. (DOCX 18 kb) [file 12957_2016_786_MOESM1_ESM.docx]

| Case | Comorbidities | Tumor Size (cm^3^) | Stage (TNM) | Procedure of Primary Site | Length of Operation (h) | Order of Operations | Blood Loss (ml) | Length of Hospital Stay (d) | Adjuvant Therapies |
| --- | --- | --- | --- | --- | --- | --- | --- | --- | --- |
| 1 | Deep vein thrombosis | 63.25 | Ⅳ | Wide resection | 6.5 | simultaneous | 800 | 10 | chemotherapy |
| 2 | None | 52.5 | Ⅳ | Partial resection | 6.2 | simultaneous | 1000 | 15 | endocrine therapy |
| 3 | None | 35 | Ⅳ | Wide resection | 5.2 | simultaneous | 600 | 11 | chemotherapy |
| 4 | None | 29.5 | Ⅳ | Partial resection | 4.2 | simultaneous | 750 | 13 | chemotherapy |
| 5 | Superficial incision infection | 42 | Ⅳ | Wide resection | 4.5 | simultaneous | 650 | 10 | chemotherapy |
| 6 | None | 95.5 | Ⅳ | Wide resection | 7.5 | simultaneous | 900 | 12 | chemotherapy |
| 7 | None | 42 | Ⅳ | Wide resection | 6.8 | simultaneous | 1050 | 14 | chemotherapy |
| 8 | None | 31.5 | Ⅳ | Wide resection | 5.6 | simultaneous | 600 | 11 | chemotherapy |
| 9 | None | 26.5 | Ⅳ | Partial resection | 5.2 | simultaneous | 750 | 12 | chemotherapy |
| 10 | None | 30 | Ⅳ | Wide resection | 4.6 | simultaneous | 800 | 13 | chemotherapy |
| 11 | None | 52.5 | Ⅳ | Wide resection | 5.5 | simultaneous | 900 | 13 | chemotherapy |
| 12 | None | 42 | Ⅳ | Partial resection | 5.8 | simultaneous | 1000 | 14 | chemotherapy |
| 13 | None | 57.75 | Ⅳ | Wide resection | 6.8 | simultaneous | 1100 | 15 | castration therapy |
| 14 | Pulmonary infection | 43.5 | Ⅳ | Partial resection | 7.2 | simultaneous | 850 | 11 | chemotherapy |
| 15 | None | 35 | Ⅳ | Wide resection | 7.5 | simultaneous | 900 | 12 | chemotherapy |
| 16 | None | 41.5 | Ⅳ | Partial resection | 5.6 | simultaneous | 750 | 10 | chemotherapy |
| 17 | None | 74.75 | Ⅳ | Wide resection | 6.5 | simultaneous | 550 | 13 | chemotherapy |
| 18 | None | 68.25 | Ⅳ | Partial resection | 6.2 | simultaneous | 700 | 11 | chemotherapy |
| 19 | None | 33.5 | Ⅳ | Wide resection | 5.5 | simultaneous | 800 | 10 | chemotherapy |
| 20 | None | 26.25 | Ⅳ | Wide resection | 6.0 | simultaneous | 850 | 13 | chemotherapy |
